# Broadened population-level frequency tuning in the auditory cortex of tinnitus patients

**DOI:** 10.1152/jn.00385.2016

**Published:** 2017-01-04

**Authors:** Kenichi Sekiya, Mariko Takahashi, Shingo Murakami, Ryusuke Kakigi, Hidehiko Okamoto

**Affiliations:** ^1^Department of Integrative Physiology, National Institute for Physiological Sciences, Okazaki, Japan;; ^2^Department of Otolaryngology, Head, and Neck Surgery, Nagoya City University Graduate School of Medical Sciences and Medical School, Nagoya, Japan; and; ^3^The Graduate University for Advanced Studies (SOKENDAI), Hayama, Japan

**Keywords:** auditory-evoked response, brain, tinnitus, frequency tuning, magnetoencephalography, MEG

## Abstract

Tinnitus is a phantom auditory perception without an external sound source and is one of the most common public health concerns that impair the quality of life of many individuals. However, its neural mechanisms remain unclear. We herein examined population-level frequency tuning in the auditory cortex of unilateral tinnitus patients with similar hearing levels in both ears using magnetoencephalography. We compared auditory-evoked neural activities elicited by a stimulation to the tinnitus and nontinnitus ears. Objective magnetoencephalographic data suggested that population-level frequency tuning corresponding to the tinnitus ear was significantly broader than that corresponding to the nontinnitus ear in the human auditory cortex. The results obtained support the hypothesis that pathological alterations in inhibitory neural networks play an important role in the perception of subjective tinnitus.

**NEW & NOTEWORTHY** Although subjective tinnitus is one of the most common public health concerns that impair the quality of life of many individuals, no standard treatment or objective diagnostic method currently exists. We herein revealed that population-level frequency tuning was significantly broader in the tinnitus ear than in the nontinnitus ear. The results of the present study provide an insight into the development of an objective diagnostic method for subjective tinnitus.

## Abstract (precis)

Although subjective tinnitus is one of the most common public health concerns that impair the quality of life of many individuals, no standard treatment or objective diagnostic method currently exists. We herein revealed that population-level frequency tuning was significantly broader in the tinnitus ear than in the nontinnitus ear. The results of the present study provide an insight into the development of an objective diagnostic method for subjective tinnitus.

subjective tinnitus, which is a phantom auditory sensation without any external sound source (Eggermont and Roberts 2004; Lockwood et al. 2002; Møller 2010), has been shown to severely deteriorate the quality of life of 1-3% of the adult population (Coles 1984; Mohamad et al. 2016; Shargorodsky et al. 2010). Pharmacological treatments (Langguth and Elgoyhen 2012; Beebe Palumbo et al. 2015), acupuncture (He et al. 2016), transcranial magnetic stimulation (Soleimani et al. 2016), deep brain stimulation (Smit et al. 2015), cognitive-behavioral therapy (Hesser et al. 2012), cochlear implantation (Ramakers et al. 2015), and sound therapy (Hoare et al. 2014; Hobson et al. 2012; Okamoto et al. 2010b; Stein et al. 2016; Wunderlich et al. 2015) have been applied to the treatment of subjective tinnitus; however, their efficiencies are still under debate (Langguth 2015). One of the main difficulties associated with the treatment of tinnitus is that its etiology has not yet been elucidated in detail.

Tinnitus appears to be frequently triggered by a sensorineural impairment in the cochlea; however, the central auditory system also appears to play an important role in the perception of tinnitus (Adjamian et al. 2009; Eggermont 2015; Elgoyhen et al. 2015; Gold and Bajo 2014; Parra and Pearlmutter 2007; Shore et al. 2016). Human neuroimaging studies using positron emission tomography (Lanting et al. 2009; Schecklmann et al. 2013), single-photon emission computed tomography (Farhadi et al. 2010; Ueyama et al. 2015), functional magnetic resonance imaging (Lanting et al. 2014), and magnetoencephalography (MEG) (Pantev et al. 1989; Weisz et al. 2005) identified subcortical and cortical activity increments in tinnitus patients. Moreover, a recent study using residual inhibition suggested that aberrant neural activity occurring in the tinnitus frequency region of the auditory cortex plays an important role in tinnitus perception (Roberts et al. 2015). These tinnitus-related modulations in neural activity appear to mainly occur in neural areas corresponding to around the hearing loss frequency. However, several studies have suggested that subjective tinnitus is not always accompanied by hearing loss (for a review, see cf. Baguley et al. 2013). Most tinnitus studies have investigated brain activity in humans or animals with hearing loss; therefore, it has not yet been clarified whether brain activity measured in these studies reflects hearing loss, subjective tinnitus, or a combination of the two (Sereda et al. 2013).

The aim of the present study is to examine the hypothesis that the subjective tinnitus sensation is associated with broadened frequency tuning within the human auditory cortex irrespective of hearing loss. To achieve this goal, we objectively measured population-level frequency tuning, which reflects inhibitory neural networks, in unilateral tinnitus patients with similar hearing threshold levels in both ears using MEG. Previous studies (Alves-Pinto et al. 2016; Okamoto et al. 2009, 2010a; Patterson et al. 1982; Sams and Salmelin 1994) succeeded in objectively measuring population-level frequency tuning in the auditory cortex by using tonal test stimuli (TS), which are presented either in isolation or embedded in band-eliminated noises (BENs). The neurons of the auditory cortex activated by TS or BEN partially overlap, and the degree of this overlap depends on the sharpness of population-level frequency tuning. The results of the present study provide an insight into the neural mechanisms responsible for tinnitus and may contribute to the development of a new strategy for objective assessments of subjective tinnitus.

## MATERIALS AND METHODS

### 

#### Participants.

Seven healthy individuals (3 females) ranging between 45 and 65 yr old (mean 54.7) participated in the present study. They had unilateral tonal tinnitus; however, their hearing thresholds obtained by means of pure tone audiometry were similar in both ears. Participants were fully informed about the study and gave written informed consent for their participation in accordance with procedures approved by the Ethics Commission of the National Institute for Physiological Sciences. All participants underwent a pure tone audiometry test and MEG measurements, while six participated in distortion product otoacoustic emissions (DPOAE) testing (Lonsbury-Martin and Martin 1990).

#### Stimuli and experimental design.

We presented a pure tone corresponding to each participant’s tinnitus frequency as a test stimulation (TS) either in isolation or embedded in BEN. TS had a duration of 500 ms with 10-ms rise and fall times. Simultaneously presented BEN was prepared as follows: from 10,000 Hz low-pass filtered white noise (sampling rate: 48,000 Hz), spectral frequency bands within 1 critical bandwidth centered at the TS frequency were eliminated. All BENs had a duration of 3,000 ms (10-ms rise and fall ramps). TS started 2,200 ms after the onset of BEN and ceased 300 ms before its offset. TS were monaurally and BENs were diotically delivered through a plastic tube with silicon earpieces fit to the participant’s ear from a speaker (KVD-200; KOBATEL, Yokohama, Japan) located outside of a magnetically shielded room. Before the initiation of MEG data acquisition, each subject’s hearing threshold for TS was assessed for each ear. The loudness of sound presentation during the MEG measurement was adjusted according to the participant’s comfort level, resulting in an intensity that was 35-40 dB higher than individual sensation levels. The total power of simultaneously presented BENs was adjusted as the participant effortlessly picked up TS embedded within BEN, resulting in a 4- to 17-dB increase in the power of BEN over TS. The sensation levels of TS and the power difference between TS and BEN were identical between the tinnitus and nontinnitus ears in each participant. To investigate the effects of BEN (“Noisy” vs. “Silent”) and the stimulated ear side (“Tinnitus” vs. “Nontinnitus”), we prepared four conditions: TS with BEN presented to the tinnitus ear (“Noisy_Tinnitus”) or nontinnitus ear (“Noisy_Nontinnitus”) and TS alone presented to the tinnitus ear (“Silent_Tinnitus”) or nontinnitus ear (“Silent_Nontinnitus”). Each MEG session consisted of 12 blocks (3 blocks per condition) of 50 trials, resulting in 600 trials (150 trials per condition). The block order was pseudorandomized among participants.

#### Data acquisition and analysis.

We obtained auditory-evoked fields with a helmet-shaped 204-channel whole head planar-type gradiometer (Vector-view, ELEKTA; Neuromag, Helsinki, Finland) in a magnetically shielded and acoustically silent room. During the MEG measurement, participants were comfortably seated upright and instructed not to move and to watch the movie on the screen to avoid paying attention to the auditory signals. The head position was monitored via a video camera. The magnetic fields measured were digitally sampled at a rate of 1,000 Hz. Epochs of data elicited by TS, including a 200-ms pre-TS-onset interval and 500-ms post-TS-onset interval, were averaged selectively for each BEN condition (“Noisy” vs. “Silent”) and each stimulated ear side (“Tinnitus” vs. “Nontinnitus”) after the rejection of artifact epochs containing field changes larger than 3 pT/cm.

In the analysis of the N1m response, which is the major component of the auditory-evoked field (for a review, see Näätänen and Picton 1987), averaged magnetic field signals were 30-Hz low-pass filtered, followed by a baseline correction relative to the 200-ms pre-TS interval. The time point of the maximal global field power, measured as the root-mean-square across all gradiometers ~100 ms after the stimulus onset, was initially identified as the N1m response. Source locations and orientations were estimated by means of two single equivalent current dipoles (one for each hemisphere) based on the grand-averaged MEG waveforms of all gradiometers for each participant. The estimated source for each hemisphere of each subject was then fixed in its location and orientation (Tesche et al. 1995), and source strengths were calculated for each BEN condition (“Noisy” vs. “Silent”) and each stimulated ear side (“Tinnitus” vs. “Nontinnitus”). In each condition and hemisphere, the N1m source strength was defined as the peak amplitude of the source strength waveform in the time interval between 75 and 225 ms.

The N1m source strengths and latencies elicited by TS in each condition were analyzed separately via repeated-measures ANOVA using BEN (“Noisy” vs. “Silent”) and EAR (“Tinnitus” vs. “Nontinnitus”) as factors.

## RESULTS

**Fig. 1.**
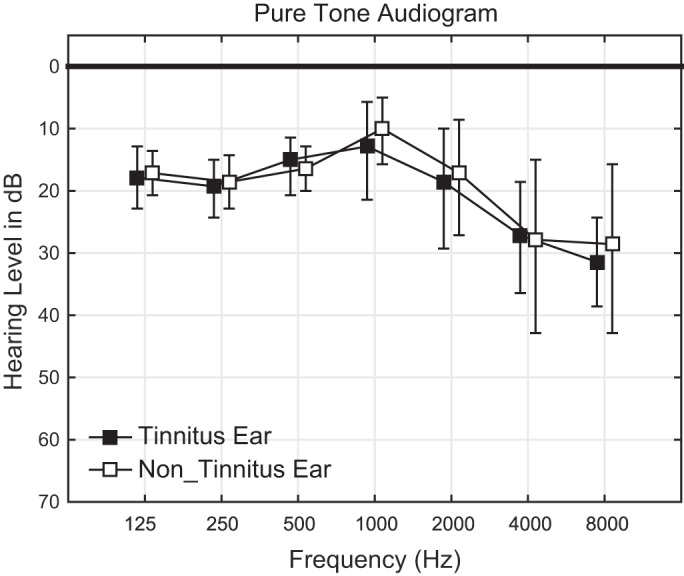
Pure tone audiograms obtained from unilateral tinnitus patients. Filled and open squares indicate the mean hearing threshold levels (dB) in the tinnitus and nontinnitus ears, respectively. Error bars denote 95% confidence intervals.

The detailed profiles of the participants are shown in Table 1. Their tinnitus side was on the left in six cases and on the right in one case, and the tinnitus frequencies obtained using a step size of 1,000 Hz were between 2,000 and 8,000 Hz (mean 5,429 Hz). The means and confidence intervals of the hearing levels obtained by pure tone audiometry were shown in Fig. 1. Paired *t*-tests revealed no significant hearing level difference between the tinnitus and nontinnitus ears at 125 Hz [(*t*_(6)_ = 0.35; *P* = 0.74], 250 Hz [*t*_(6)_ = 0.55; *P* = 0.60], 500 Hz [*t*_(6)_ = −1.00; *P* = 0.36], 1,000 Hz [*t*_(6)_ = 1.19; *P* = 0.28], 2,000 Hz [*t*_(6)_ = 1.00; *P* = 0.36], 4,000 Hz [*t*_(6)_ = −0.21; *P* = 0.84], or 8,000 Hz [*t*_(6)_ = 0.64; *P* = 0.55]. The means ± SD of hearing levels at the tinnitus frequency were 26.4 ± 14.6 dB in the tinnitus ear and 28.6 ± 21.9 dB in the nontinnitus ear, and a paired *t*-test revealed no significant difference between them [*t*_(6)_ = −0.55; *P* = 0.60]. Hearing levels were similar between the tinnitus and nontinnitus ears in all participants. DPOAE testing around the tinnitus frequency was performed on six participants (except for participant 5 in Fig. 1). Data from one participant (*participant 3* in Fig. 1) exhibited very low signal-to-noise ratios in both the tinnitus and nontinnitus ears and was excluded from further statistical analyses. A paired *t*-test revealed no significant differences in DPOAE levels between the tinnitus and nontinnitus ears (*t*_(4)_ = −1.10; *P* = 0.33).

**Table 1.** Participant characteristics

					Mean Hearing Level, dB		Hearing Level at Tinnitus Frequency, dB	
Participant	Age, yr	Gender	Tinnitus Side	Tinnitus Pitch	Tinnitus Ear	Nontinnitus Ear	Tinnitus Ear	Nontinnitus Ear
1	59	M	Right	8,000	13.8	13.8	25	20
2	58	F	Left	2,000	10.0	12.5	5	10
3	48	F	Left	4,000	10.0	8.8	20	10
4	45	M	Left	4,000	16.2	16.2	20	20
5	65	M	left	8,000	28.8	31.3	40	60
6	61	M	Left	4,000	36.2	35.0	50	60
7	49	F	Left	8,000	11.2	7.5	25	20

Mean hearing levels were obtained by averaging hearing thresholds at 500, 1,000, 2,000, and 4,000 Hz. M, male; F, female.

**Fig. 2.**
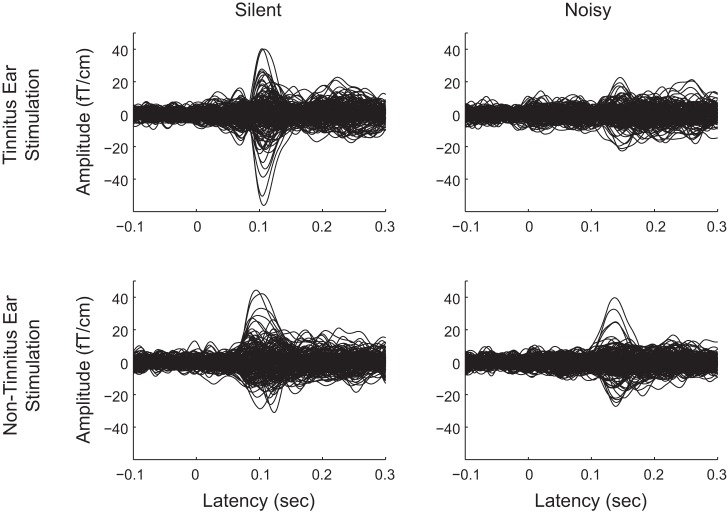
Auditory-evoked magnetic fields of one representative participant. *Top* and *bottom*: graphs represent auditory-evoked fields elicited by the tinnitus ear stimulation and nontinnitus ear stimulation, respectively. *Left* and *right*: columns represent auditory-evoked fields elicited in silence and within band-eliminated noise, respectively.

**Fig. 3.**
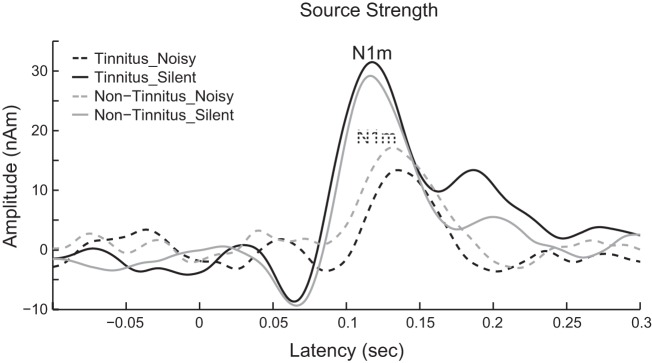
Means of source strength waveforms across all participants (*n* = 7) and hemispheres. Solid lines represent the “Silent” condition and dashed lines represent the “Noisy” condition. Thin black lines represent the tinnitus ear stimulation and thick gray lines represent the nontinnitus ear stimulation.

Clearly identifiable N1m responses were obtained from all participants. Exemplary MEG data are shown in Fig. 2. After artifact rejection, a sufficient number of trials remained in each condition to be used in the auditory-evoked N1m analysis (means ± SD; “Noisy_Tinnitus” 147.6 ± 1.4, “Silent_Tinnitus” 149.3 ± 0.6, “Noisy_Nontinnitus 147.4 ± 1.6, “Silent_Nontinnitus” 147.9 ± 1.4). The locations of the equivalent current dipoles corresponding to the N1m responses did not show any systematic difference between the “Noise” and “Silent” conditions, as described in previous studies (Lagemann et al. 2012; Okamoto et al. 2010a, 2011; Sams and Salmelin 1994). The grand-averaged N1m cortical source waveforms across all participants (*n =* 7) and hemispheres ranging between −200 and +500 ms are shown in Fig. 3. This figure demonstrates clear N1m responses that peaked ~100 ms after the onset of TS for the “Silent” condition. N1m responses showed smaller and later N1m peaks in the “Noisy” condition than in the “Silent” condition. The goodness-of-fit of the underlying dipolar model for the dipole estimation was in the range of 86.5-96.4% (92.1 ± 1.3), confirming the adequacy of the selected equivalent current dipole approach.

**Fig. 4.**
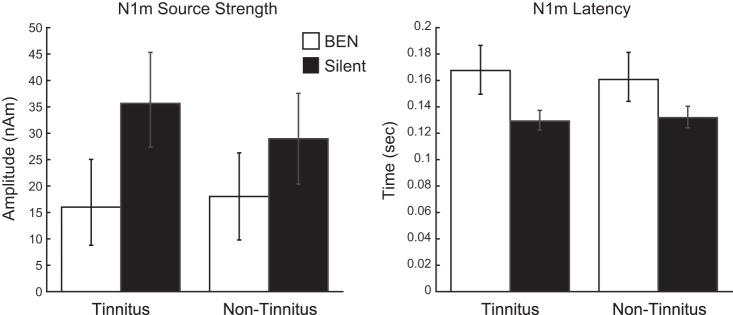
Group means (*n* = 7) of N1m source strengths (*left*) and latencies (*right*) obtained by tinnitus and nontinnitus ear stimuli. Error bars denote the 95% confidence intervals obtained by boot-strap resampling tests (iteration = 100,000). Open and Filled bars denote the “Noisy” and “Silent” conditions, respectively.

The mean N1m source strength and N1m latency in each BEN and each EAR condition are presented with 95% confidence intervals in Fig. 4. The repeated-measures ANOVA applied to N1m source strengths identified the significant main effect of BEN [*F*_(1,6)_ = 7.67; *P* = 0.032], as well as a significant interaction between BEN and EAR [*F*_(1,6)_ = 16.61; *P* = 0.007]. N1m source strength was significantly larger in the “Silent” condition than in the “Noisy” condition. The effects of BEN on N1m source strength increased when TS was presented to the tinnitus ear.

The repeated-measures ANOVA applied to N1m latency identified the significant main effect of BEN [*F*_(1,6)_ = 29.17; *P* = 0.002], whereas a significant interaction was not observed between BEN and EAR. N1m responses were significantly delayed by the presence of BEN; however, EAR did not affect the latency of N1m.

## DISCUSSION

We herein demonstrated that decrements in N1m responses due to BEN were more prominent when TS was presented to the tinnitus ear than to the nontinnitus ear (see Figs. 3 and 4). These results suggest that population-level frequency tuning was significantly broader when sounds were delivered to the tinnitus ear than to the nontinnitus ear, supporting the hypothesis that inhibitory neural mechanisms become weaker around the tinnitus frequency in the afferent auditory pathway. Our participants did not suffer from hearing loss and had similar hearing levels between both ears. Therefore, the results obtained reflect neural activity related to the existence of subjective tinnitus and not to hearing loss.

**Fig. 5.**
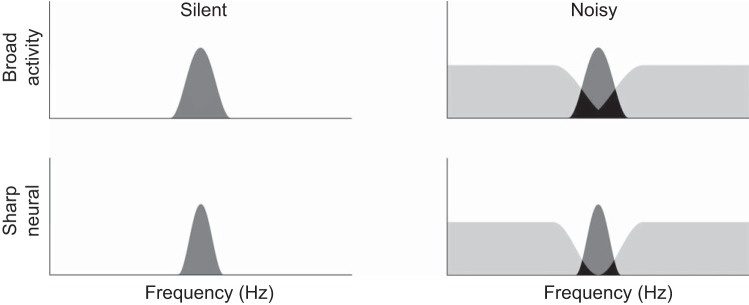
Schematic figures of population-level frequency tuning. *Left* and *right*: columns represent neural activity elicited by a test stimulus without and with band-eliminated noise (BEN). *Top* and *bottom*: represent broad and sharp population-level frequency tuning, respectively. The 3 different colored areas represent 3 distinct neural groups: *1*) neurons activated by BEN (light gray areas), *2*) neurons activated by a test stimulus (dark gray areas), and *3*) neurons activated by both BEN and a test stimulus (black areas).

To objectively investigate population-level frequency tuning in the human auditory cortex, we measured auditory-evoked fields utilizing the tinnitus frequency as TS and simultaneously presented BEN as a continuous masking sound, similar to previous studies (Okamoto et al. 2007, 2010a; Sams and Salmelin 1994). Neural activity elicited by the combination of TS and BEN has been categorized into three groups, as shown in Fig. 5: *1*) neural activity evoked solely by TS, *2*) neural activity evoked solely by BEN, and *3*) neural activity elicited by both TS and BEN. The N1m responses obtained in the present study appear to represent the summation of neural *groups 1* and *3* in the “Silent” condition and *group 1* in the “Noisy” condition because neural *group 3* had already been activated by preceding BEN when TS was presented. We found that BEN decreased N1m source strengths more strongly when TS was presented to the tinnitus ear than to the nontinnitus ear (Fig. 4). The presentation of BEN to the tinnitus ear appeared to increase the number of overlapping neural populations (neural *group 3*), and, thus, a smaller neural group (neural *group 1*) was newly activated by the subsequent onset of TS. These results indicate that auditory neural activity corresponding to the tinnitus ear was characterized by broader population-level frequency tuning than that corresponding to the nontinnitus ear.

The broadened population-level frequency tuning of neural activity corresponding to the tinnitus ear in the present study implied pathological alterations in inhibitory neural networks and supported the hypothesis that reduced inhibitory neural networks in the central auditory pathway play an important role in the emergence and maintenance of subjective tinnitus symptoms (Diesch et al. 2004, 2010; Kral and Majernik 1996). Damage to excitatory neurons, such as the loss of inner hair cells, elevates the hearing threshold; however, pathological alterations in inhibitory neural networks may not influence the hearing threshold but may instead lead to broadened frequency tuning in the auditory system and the emergence of tinnitus. The participants in the present study had unilateral tinnitus but had similar hearing and DPOAE levels between the tinnitus and nontinnitus ears. Moreover, the MEG results showed that N1m source strengths elicited in the tinnitus ear were larger in the “Silent” condition and smaller in the “Noisy” condition than those in the nontinnitus ear. These results support the hypothesis that inhibitory neural networks corresponding to the tinnitus ear are more pathologically impaired than the excitatory neurons in tinnitus patients. The inhibitory neural networks within the auditory cortex appear to contribute to sharpening frequency tuning through the suppression of excitatory neural activity corresponding to neighboring frequencies (Wehr and Zador 2003) and enabling the context-dependent modulation of sensory perception during acoustic behaviors (Kuchibhotla et al. 2017). Previous studies (Aizenberg et al. 2015; Natan et al. 2015) demonstrated that parvalbumin-positive interneurons nonspecifically inhibited neural activity in the primary auditory cortex of mice, whereas somatostatin-positive interneurons reduced excitatory neural activity in a frequency-specific manner. In the present study, the larger N1m source strengths elicited by the tinnitus ear stimulation in the “Silent” condition imply a decrease in parvalbumin-positive interneuron activity, whereas the smaller N1m source strength elicited by the tinnitus ear stimulation in the “Noisy” condition appears to reflect the activity of somatostatin-positive interneurons. Pathological alterations in parvalbumin-positive interneurons and somatostatin-positive interneurons as well as subsequent multiple excitatory-inhibitory neural interactions may lead to the emergence and maintenance of tinnitus perception.

Focused auditory attention not only amplifies auditory-evoked neural responses, it also sharpens population-level frequency tuning in the human auditory cortex (Bidet-Caulet et al. 2007; Engell et al. 2016; Okamoto et al. 2007, 2009). In the present study, participants were distracted from the auditory modality during the MEG measurement. However, a previous study (Cuny et al. 2004) demonstrated that auditory attention was automatically directed to the tinnitus ear in unilateral tinnitus patients. Therefore, the participants in the present study may also have involuntarily directed their attention to the tinnitus ear and, as a consequence, sharpened population-level frequency tuning. However, the results obtained demonstrated sharper frequency tuning in the nontinnitus ear stimulation than in the tinnitus ear stimulation. Therefore, it is less likely that involuntarily directed attention to the tinnitus ear caused the present results.

In conclusion, we demonstrated by means of MEG that the population-level frequency tuning of neural activity related to the tinnitus ear was broadened in unilateral tinnitus patients who had similar hearing levels in both ears. These results suggest that pathological alterations in inhibitory neural networks play an important role in the perception of tinnitus. Broadened population-level frequency tuning in the auditory cortex may be easily compensated for by auditory focused attention, which is inevitable in most clinical auditory assessments. Our results may contribute to the development of an objective assessment of subjective tinnitus perception, which is currently very difficult.

## GRANTS

This work has been supported by the Japan Society for the Promotion of Science for Young Scientists (26861426).

## DISCLOSURES

No conflicts of interest, financial or otherwise, are declared by the authors.

## AUTHOR CONTRIBUTIONS

K.S., M.T., and H.O. performed experiments; K.S. and H.O. analyzed data; K.S. and H.O. interpreted results of experiments; K.S. and H.O. prepared figures; K.S. and H.O. drafted manuscript; K.S., M.T., S.M., R.K., and H.O. edited and revised manuscript; K.S., M.T., S.M., R.K., and H.O. approved final version of manuscript; H.O. conceived and designed research.
